# Neural correlates of mating system diversity: oxytocin and vasopressin receptor distributions in monogamous and non-monogamous *Eulemur*

**DOI:** 10.1038/s41598-021-83342-6

**Published:** 2021-02-12

**Authors:** Nicholas M. Grebe, Annika Sharma, Sara M. Freeman, Michelle C. Palumbo, Heather B. Patisaul, Karen L. Bales, Christine M. Drea

**Affiliations:** 1 Department of Evolutionary Anthropology, Duke University, Durham, NC USA; 2 Department of Psychology, California National Primate Research Center, University of California-Davis, Davis, CA USA; 3 Department of Biology, Utah State University, Logan, UT USA; 4 Department of Behavioral Neuroscience, Oregon Health and Science University, Portland, OR USA; 5 Department of Biological Sciences, North Carolina State University, Raleigh, NC USA

**Keywords:** Anthropology, Social evolution, Social behaviour, Social neuroscience, Brain, Psychology

## Abstract

Contemporary theory that emphasizes the roles of oxytocin and vasopressin in mammalian sociality has been shaped by seminal vole research that revealed interspecific variation in neuroendocrine circuitry by mating system. However, substantial challenges exist in interpreting and translating these rodent findings to other mammalian groups, including humans, making research on nonhuman primates crucial. Both monogamous and non-monogamous species exist within *Eulemur*, a genus of strepsirrhine primate, offering a rare opportunity to broaden a comparative perspective on oxytocin and vasopressin neurocircuitry with increased evolutionary relevance to humans. We performed oxytocin and arginine vasopressin 1a receptor autoradiography on 12 *Eulemur* brains from seven closely related species to (1) characterize receptor distributions across the genus, and (2) examine differences between monogamous and non-monogamous species in regions part of putative “pair-bonding circuits”. We find some binding patterns across *Eulemur* reminiscent of olfactory-guided rodents, but others congruent with more visually oriented anthropoids, consistent with lemurs occupying an ‘intermediary’ evolutionary niche between haplorhine primates and other mammalian groups. We find little evidence of a “pair-bonding circuit” in *Eulemur* akin to those proposed in previous rodent or primate research. Mapping neuropeptide receptors in these nontraditional species questions existing assumptions and informs proposed evolutionary explanations about the biological bases of monogamy.

## Introduction

“Undoubtedly there are numerous molecular and neurobiological pathways that could evolve to support pair-bond formation between mates, and different species may have achieved similar behaviors through a process of convergent evolution involving different circuits… Nevertheless, it is intriguing to consider the possibility that similar mechanisms may underlie the formation of pair bonds in both humans and rodents.” –Young and Wang (2004, p. 1052).

Behavioral biologists are keenly interested in the evolved mechanisms that underlie diversity in social systems. Oxytocin and vasopressin, two closely related neuropeptides, have been promising hormonal candidates in the study of social systems due to their socioregulatory functions spanning a range of behavior in a wide variety of taxa^1^. Beyond their conserved physiological roles, key behavioral and cognitive processes modulated by these nonapeptides include pair bonding and mating^2–5^, parental care^6,7^, stress coping^8,9^, aggression^10,11^, social reward^12^, and social recognition^13,14^. Many hypotheses regarding the mechanisms by which oxytocin and vasopressin modulate attachment behavior, and even mating systems, stem from comparative rodent work examining underlying neural circuits. Hypotheses arising from this work posed the exciting possibility that mechanisms of neuropeptide function might generalize to humans; nevertheless, primate models that would provide important, evolutionarily relevant tests of these mechanisms have been underutilized^15,16^. Here, we examine neuroanatomical distributions of oxytocin and vasopressin receptors for the first time in strepsirrhine primates, taking advantage of the distinct interspecific variation in social system within a single genus to test the influential hypothesis that differences in receptor distributions reflect differences in mating system.

Only one type of oxytocin receptor, the G protein-coupled receptor OXTR, has been characterized thus far, but vasopressin has three known receptors (AVPR1a, AVPR1b and AVPR2;^17^). Of these receptors, AVPR1a is present centrally and is thought to mediate the majority of vasopressin’s social effects^17^. In foundational neuroanatomical studies on the *Microtus* genus of voles, researchers used in vitro receptor autoradiography to examine both OXTR and AVPR1a. Voles emerged as exceptional comparative models because both monogamous and non-monogamous species exist within the same genus, allowing researchers to examine neurobiological differences between phylogenetically proximate, yet socially divergent species. While the neural distributions of vasopressin and oxytocin immunoreactive fibers are relatively conserved across vertebrates^1^, receptor distributions can vary substantially between even closely related species. Indeed, researchers found striking species-level differences in the distribution of OXTR and AVPR1a between monogamous prairie voles (*Microtus ochrogaster*) and promiscuous montane and meadow voles (*Microtus montanus* and *Microtus pennsylvanicus*, respectively)^18–21^, linking these differences in neuropeptide receptor distributions to interspecific differences in the ability to form pair bonds^4,18^. This approach has since been broadly applied to examine the evolved functions of oxytocin and vasopressin in rodent social behavior (e.g.^22,23^) and has established rodent models as central to understanding oxytocin’s purportedly conserved social functions across mammals, including humans^4,24,25^.

Developments within the field of oxytocin and vasopressin research have also revealed substantial challenges to the interpretation and translation of findings from rodent models to other mammalian groups. Prairie voles exhibit substantial diversity in their mating tactics^26^ and their central distribution patterns of nonapeptide receptors^27–29^. Reducing any species’ socioecology and neurobiology to sets of strictly canalized components might oversimplify the underlying mechanisms and limit insights. More generally, behavioral endocrinologists and neuroscientists have long raised concerns about the field’s reliance on a small set of model organisms^30–32^. Additionally, research identifying unique aspects of human neurobiology^33,34^ challenges the potential translatability of rodent models. Nonhuman primates might thus serve as valuable bridges from rodent to human biology and sociality^15,16^.

The *Eulemur* genus of strepsirrhine primates represents a unique and powerful test system for this research area for two reasons. First, with regard to their morphology and sensory adaptations, lemurs are seen as occupying a ‘transitional’ evolutionary niche between rodents and the more-often studied anthropoid primates^35,36^. Features shared with rodents include enhanced olfactory reliance, acuity, and chemical communication, including a functional vomeronasal organ; features shared with haplorhine primates include forward-facing eyes and visual elaboration^35^. Despite being our most distant primate relatives, lemurs are approximately only half the genetic distance away from humans as are rodents^37^. Second, and crucially, *Eulemur* is the sole primate analogue to *Microtus* in terms of containing both monogamous and non-monogamous species in a single genus. The 12 extant species of *Eulemur* are all cathemeral, arboreal, and seasonal breeders; they are generally frugivorous and sympatric; and they are collectively found in almost all forest habitats across Madagascar^38^. Phylogenetic reconstructions have revealed group-living (with male dispersal and female philopatry) as the ancestral form of social organization in this clade^39^). Social monogamy and pair-bonding (which need not entail genetic monogamy)^40^, a year-round arrangement whereby a male–female pair lives in a small family group, defends a shared territory via mutual scent-marking, and jointly cares for young across several seasons, has evolved either once or twice, giving rise to *E. rubriventer* and *E. mongoz* as the two monogamous species in this genus^39,41–43^. All other species are non-monogamous, live in larger social groups, and exhibit varying degrees of promiscuous mating; they also lack the behavioral signatures of social pair-bonds seen in monogamous species^39,42^. Along with evidence of behavioral bifurcation, phylogenetic evidence of recent species divergence in *Eulemur*^39^ supports a consideration of two distinct categories of social systems across the genus. Thus, *Eulemur* presents us with the opportunity to examine how a conspicuous split in mating systems between closely related primate species is predicted by neuropeptide circuitry.

Because the neurobiology of the oxytocin and vasopressin systems has yet to be characterized in any strepsirrhine primate, we begin with a broad ‘discovery’ aim (1). Across species, we expect to find conserved binding to both neuropeptide receptors in certain specific regions. Parallel to the findings of OXTR/AVPR1a expression along regions of the olfactory pathway in rodents^19,20,44^, OXTRs in regions of visual processing and attention have consistently been found in anthropoid primates^45^. In strepsirrhines, we expect to find binding in nuclei involved in both perceptual modalities. Additionally, in anthropoid primates, AVPR1a generally has been found to be more widely distributed than OXTRs^46–48^—we predict a similar pattern in strepsirrhines.

With foundational neuroanatomical information in hand, we address our more targeted aim (2) to test variation in these receptors as a function of mating system. We investigate whether binding patterns in the brains of monogamous versus non-monogamous *Eulemur* differ in several regions of hypothesized “pair-bonding circuits”. Based on correlational and experimental evidence in voles^4,18,19^ linking neuropeptide receptor expression in specific nuclei to pair-bond formation, key OXTR regions include the medial amygdala, nucleus accumbens, and prefrontal cortex, and key AVPR1a regions include the ventral pallidum, lateral septum, and bed nucleus of the stria terminalis. Based on reported neuropeptide receptor distributions and patterns of central glucose uptake upon partner separation in monogamous coppery titi monkeys (*Plecturocebus cupreus*)^49^, additional key regions for either OXTR or AVPR1a include the hippocampus, lateral septum, and central amygdala. Examining differences in these predicted regions as a function of mating system, as well as any additional differences specific to *Eulemur*, will provide a powerful test of the role of neuropeptide receptor organization in predicting social diversity.

## Methods

### Specimens

**Figure 1:**
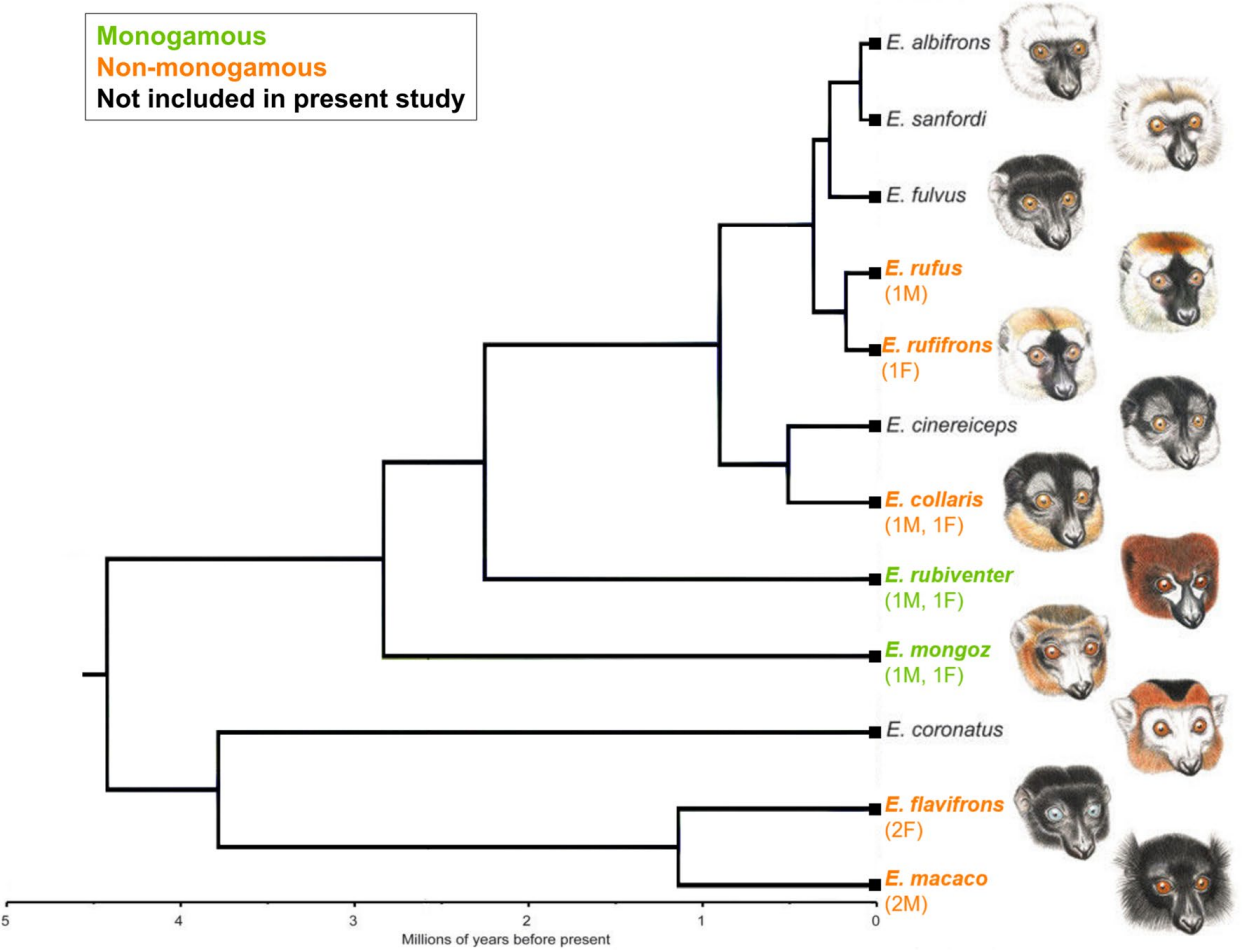
The mating system classification of seven *Eulemur* species at the Duke Lemur Center and their phylogenetic relationships, adapted from^39,51^. The number and sex of specimens from each focal species is denoted in parentheses. The source figure^51^ is published under a creative commons attribution license.

Frozen, unfixed brain specimens derived from 12 individual *Eulemur* subjects (6 M, 6 F), representing two distinct mating systems among seven closely related species (see Fig. 1 for numbers of specimens per sex and species). Members of *Eulemur* range from vulnerable to critically endangered^50^. The specimens were thus obtained from the tissue bank of the Duke Lemur Center (DLC) in Durham, NC, which is the only facility outside of Madagascar to house and/or breed several of these species. All individuals whose brains were obtained had been housed socially, primarily in adult male–female pairs, until their death from natural causes or veterinary euthanization (all for non-neurological reasons; age of death ranged from 16.6 to 34.0 years of age).

Of the 12 specimens, 11 were hemispheres, and one was a whole brain. Owing to variability in location of the midline bisections, and tissue integrity around the edges, some brain regions (such as the midline thalamic nuclei and reticulotegmental nucleus; see below) were either absent or non-quantifiable in some specimens. Additionally, the olfactory bulbs and brainstem nuclei (such as the inferior olive and spinal trigeminal nucleus) were only quantifiable in a subset of subjects (see below).

### Tissue preparation

We blocked brain specimens coronally on dry ice, before wrapping them tightly in aluminum foil and storing them at − 80 °C until sectioning. We brought hemisphere blocks up to − 20 °C for sectioning at 20 µm on one of two cryostats. We mounted tissue sections on SuperFrost Plus slides (Brain Research Labs, Newton, MA) and stored them in a sealed slide box with a desiccant packet at − 80 °C until their use in receptor autoradiography.

### Receptor autoradiography

For receptor autoradiography, we used a competitive oxytocin and vasopressin receptor binding protocol developed and optimized for primate tissue by Freeman et al*.*^46^ in rhesus macaques (*Macaca mulatta*) and validated to selectively reveal OXTR or AVPR1a binding sites in postmortem brain tissue from common marmosets (*Callithrix jacchus*;^45^), coppery titi monkeys [^47^], and humans^48,52^. After lightly fixing the tissue sections in 0.1% paraformaldehyde and rinsing with Tris buffer, we incubated them with either the OXTR radioligand ^125^I-ornithine vasotocin analogue (^125^I-OVTA; PerkinElmer, Waltham, MA) or the AVPR1a radioligand ^125^I-linear vasopressin antagonist (^125^I-LVA; PerkinElmer, Waltham, MA). We co-incubated sets of three adjacent sections in three different conditions: (i) 50 pM radioligand alone, (ii) 50 pM radioligand plus 1 nM SR49059 (Tocris, Minneapolis, MN), an AVPR1a antagonist, and (iii) 50 pM radioligand plus 100 nM ALS-II-69 (donated by ALS; see^53^), an OXTR antagonist. Accordingly, set (i) could be compared to sets (ii) and (iii) to show regions of selective binding. After incubation, we washed the slides with Tris buffer, dipped them in ddH_2_O, air dried them, and exposed them to BioMax MR film (Kodak, Rochester, NY) for four days with a set of ten^125^I autoradiographic standards (American Radiolabeled Chemicals, St. Louis, MO). After film development, we quantified receptor density directly from films without image enhancement.

Because no labelled brain atlas exists for any *Eulemur* species (or any member of the *Lemuridae* family), we delineated brain regions for image analysis by counterstaining slides for acetylcholinesterase (AChE) following a modified protocol from Lim et al.^20^ that has been shown to amplify signal in tissue previously used for receptor autoradiography.

We quantified the optical binding density (OBD) of the autoradiogram images on a light box with MCID Core Digital Densitometry software (Cambridge, UK). First, we determined a flat field correction for luminosity. Then, we loaded the optical binding values from the set of^125^I autoradiographic standards into the software and used them to generate a standard curve from which OBD values of brain regions of interest could be interpolated. To determine neuroanatomical landmarks and identify regions, we compared images to the sets of AChE counterstained slides, as well as to two atlases of rhesus macaque brains (^54,55^; www.brainmuseum.org) and an atlas of the adult human brain^56^. We made three separate measurements per brain region with identifiable OXTR/AVPR1a binding.

### Analyses

Our statistical analyses proceeded in three stages. First, we validated the competitive binding protocol using paired *t*-tests to compare results from each of the ‘competitor binding’ conditions to the ‘radioligand alone’ condition, in four representative regions as well as across all measured regions. In these analyses, we considered measurements from monogamous and non-monogamous animals together. We next performed Welch's *t*-tests to identify regions with appreciable selective binding of OXTR (^125^I-OVTA + SR49059) or AVPR1a (^125^I-LVA + ALS-II-69). Results in this section are presented as mean ± SEM estimated disintegrations per minute per milligram (dpm/mg). Lastly, to examine differences as a function of mating system, we used linear mixed models that contained replicate OBD measurements nested within individual animals as a random effect. These analyses included sex as a factor in the mixed model; however, the exclusion of sex had no substantive effect on any results presented below, indicating a lack of significant differences in binding profiles between the sexes. We performed separate models for individual regions that had either (a) been previously implicated as key areas for rodent and/or primate pair bonding, or (b) showed dense neuropeptide binding in our exploratory analyses. We report results for mating system differences as effect sizes in Cohen’s *d*, with positive values of *d* representing greater binding in specimens from monogamous lemurs. The data and corresponding R code needed to reproduce our results are publicly available at https://osf.io/rymz5/.

## Results

### Selectivity of radioligands

The radioligands^125^I-OVTA and^125^I-LVA produced distinct patterns of binding in *Eulemur* brains (Fig. S1). As in anthropoids, strepsirrhine brains required competitive binding with the AVPR1a antagonist to allow accurately identifying regions of OXTR binding. At the concentration used in our assay,^125^I-OVTA labelled both OXTR and AVPR1a. Both the AVPR1a antagonist, SR49059, and the OXTR antagonist, ALS-II-69, significantly reduced^125^I-OVTA binding in the central amygdala (CeA), nucleus accumbens (NAcc), and spinal trigeminal nucleus (Sp5) (Table S1). In contrast, the AVPR1a antagonist significantly reduced^125^I-LVA binding in the CeA, Sp5, and primary visual cortex (V1), whereas the OXTR antagonist did not reduce^125^I-LVA binding in these regions (Table S2). This selective reduction in^125^I-LVA binding by the AVPR1a antagonist showed that^125^I-LVA binds selectively to AVPR1a and not to OXTR in *Eulemur* species, while^125^I-OVTA appears to be able to bind to both receptor subtypes in some regions. Alternatively, SR49059 and ALS-II-69 may have different affinities for *Eulemur* OXTR and AVPR1a at the concentrations used in this study. Fig. S1 shows the overall efficacy of the antagonists for displacing radioligand binding. Based on these results, below we present values for sections incubated with both the radioligand and the opposing receptor antagonist:^125^I-OVTA + SR49059, and^125^I-LVA + ALS-II-69.

### OXTR distribution Across *Eulemur* brains

**Figure 2:**
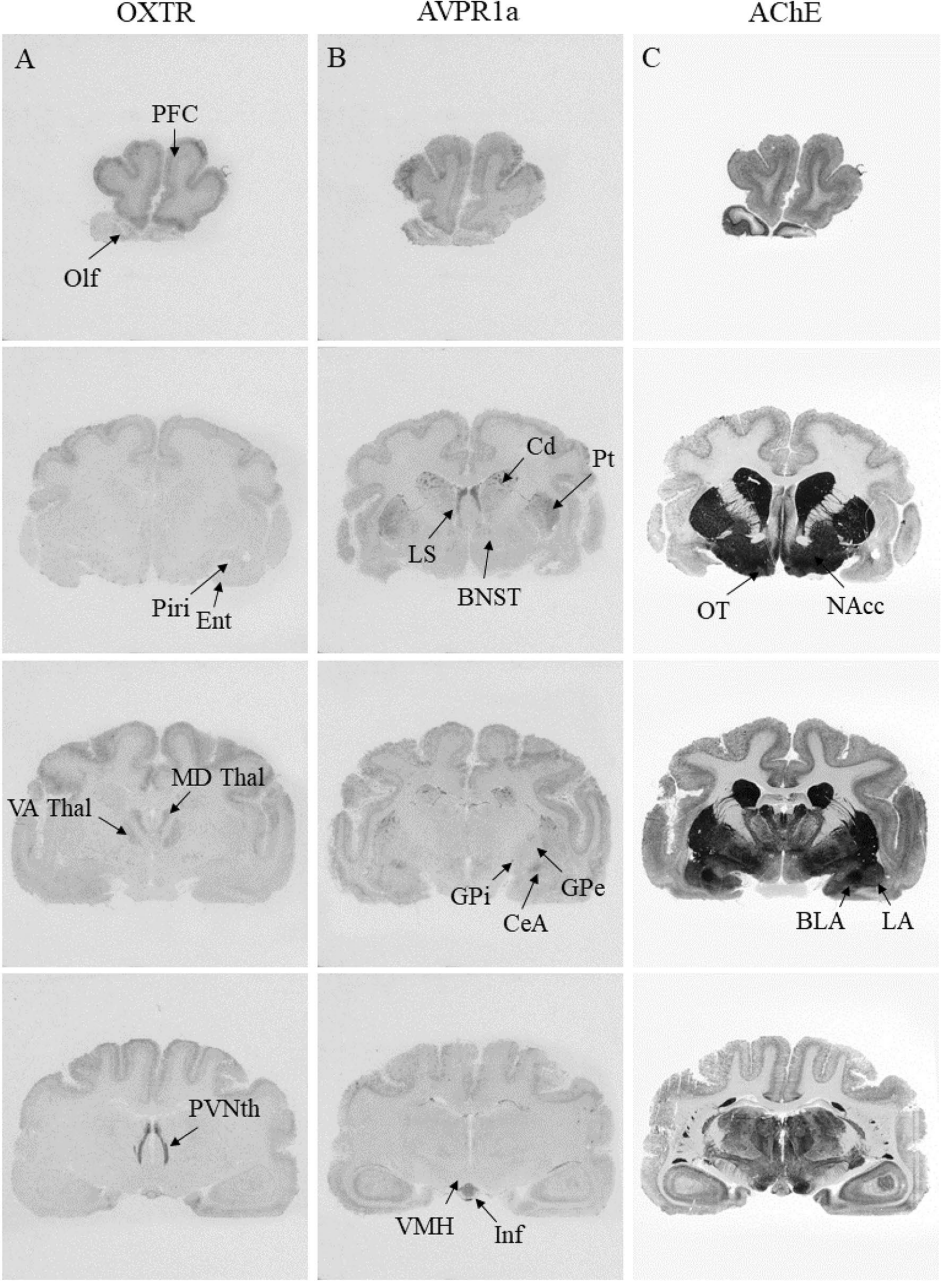

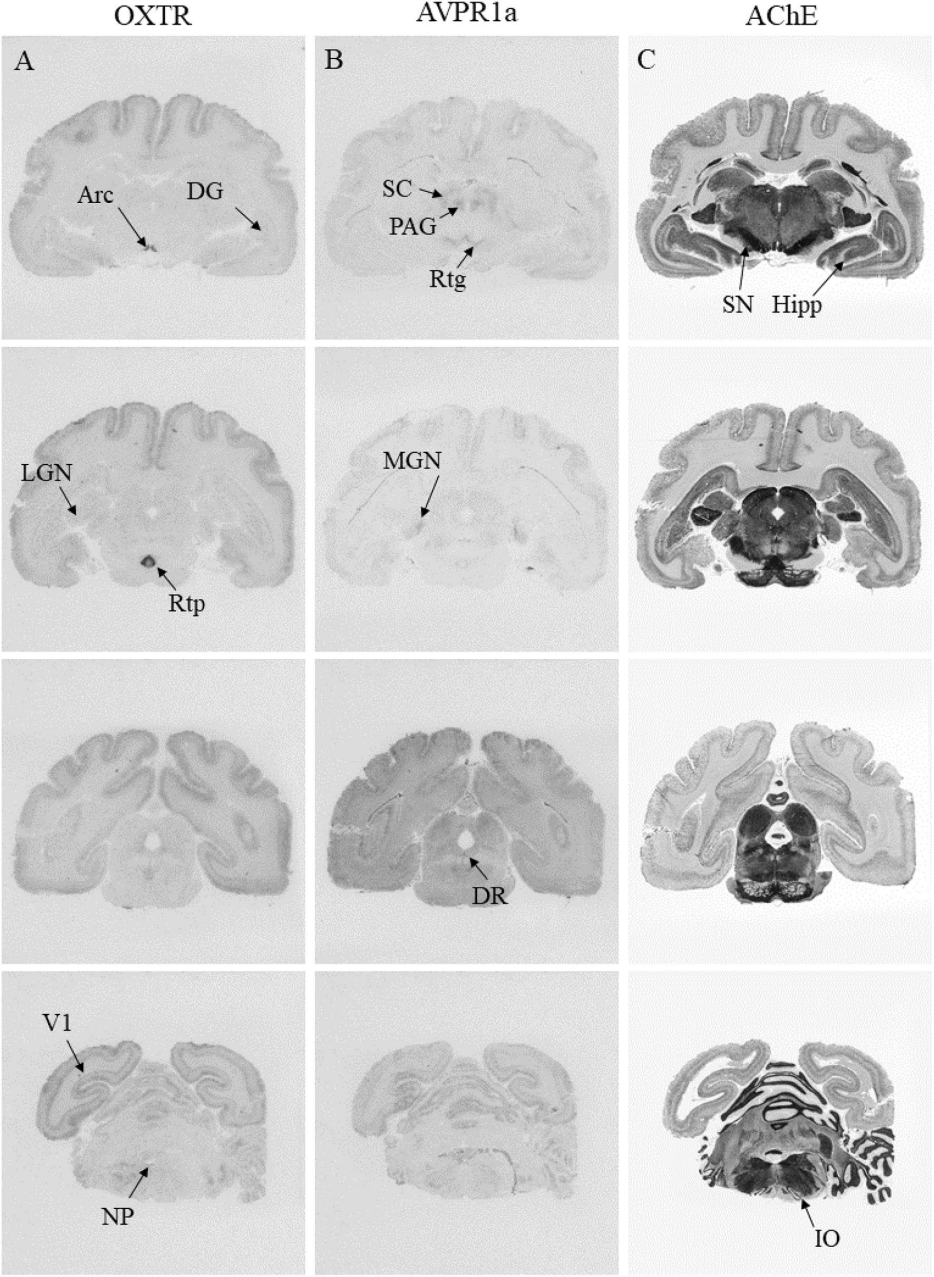
Distribution of OXTR (**A**) and AVPR1a (**B**) in sequential coronal sections from the brain of one representative non-monogamous *Eulemur* individual (*E. macaco)*, aligned with acetylcholinesterase (AChE) counterstain (**C**). Panels 1–2.

**Figure 3:**
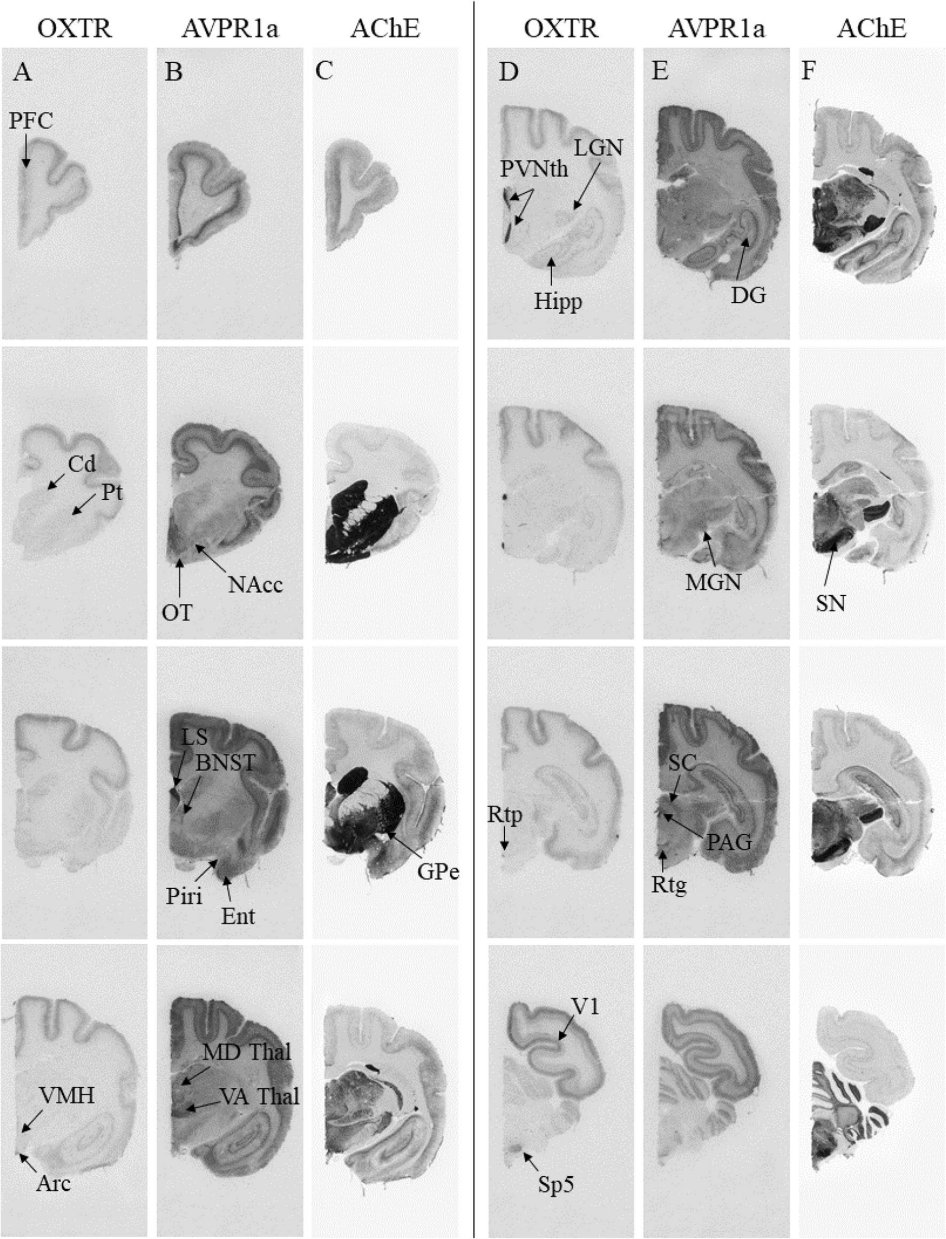
Distribution of OXTR (**A**, **D**) and AVPR1a (**B**, **E**) in sequential coronal sections from the brain of one representative monogamous *Eulemur* individual (*E. rubriventer)*, aligned with acetylcholinesterase (AChE) counterstain (**C**, **F**).

**Figure 4:**
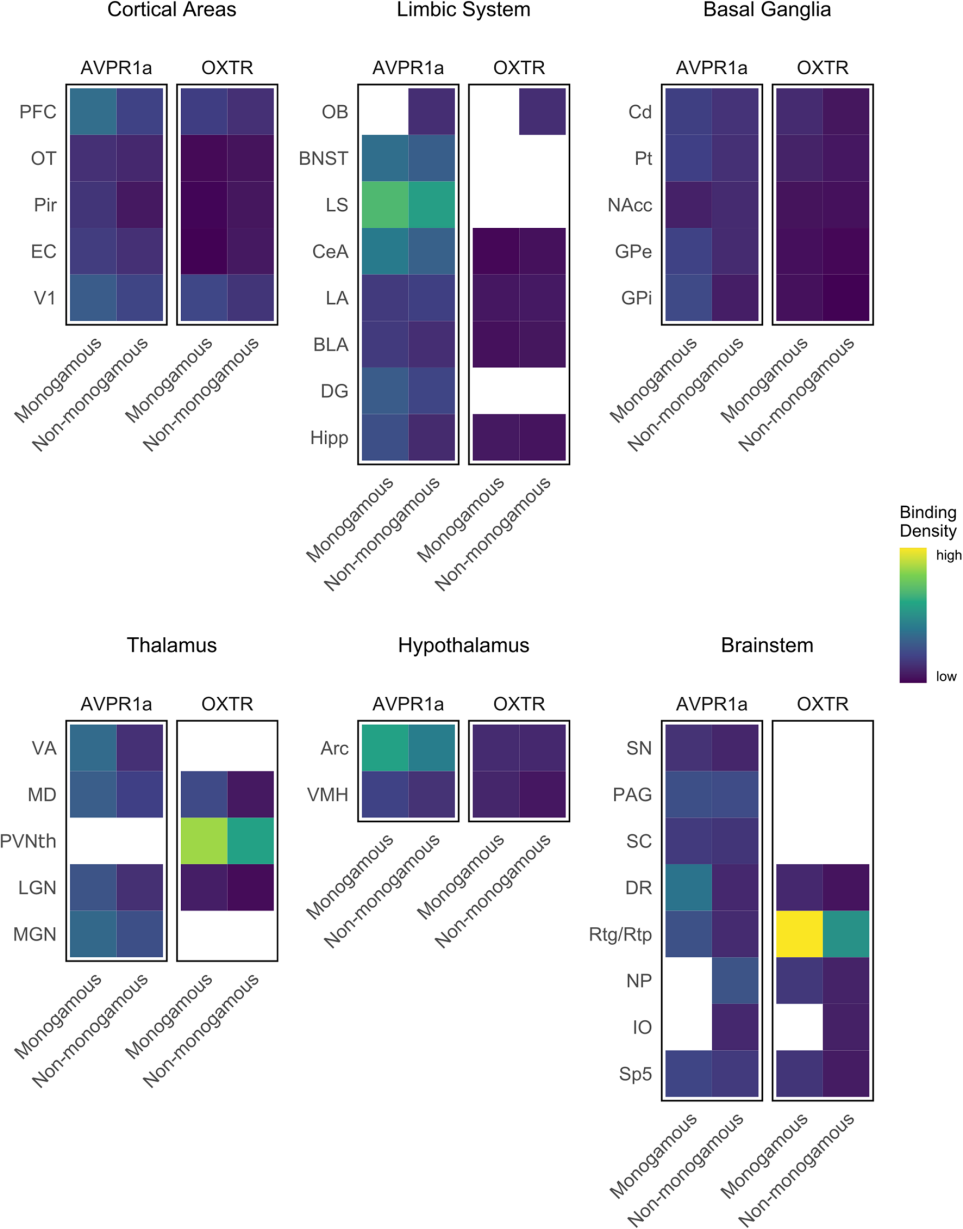
Summary of relative binding densities by brain region in *Eulemur* species. Columns depict average binding density across brain specimens from monogamous and non-monogamous species, respectively. Blank cells indicate no measurement available for radioligand binding in that region.

Strong^125^I-OVTA binding in the presence of the AVPR1a antagonist was restricted to few areas (Figs. 2A, 3A,D), including the paraventricular nucleus of the thalamus (PVNth; 343.48 ± 39.17), V1 (86.66 ± 17.33), prefrontal cortex (PFC; 76.82 ± 12.50), mediodorsal thalamus (MD Thal; 72.66 ± 32.97), and olfactory bulb (Olf; 61.99 ± 26.88; this region was only present in specimens from non-monogamous species; Figs. 2, 4). We observed modest OXTR binding in the hypothalamus (arcuate nucleus [Arc]: 55.00 ± 19.40; ventromedial hypothalamus [VMH]: 34.36 ± 12.53), striatum (caudate [Cd]: 37.58 ± 7.56; putamen [Pt]: 33.43 ± 6.26; NAcc: 19.20 ± 6.31), and assorted brainstem nuclei (nucleus prepositus [NP]: 52.07 ± 8.37; Sp5: 43.86 ± 9.51). We found low levels of binding in the olfactory tubercle (OT), piriform cortex (Pir), entorhinal cortex (EC), globus pallidus external and internal segments (GPe / GPi), various amygdalar nuclei (CeA; LA; BLA), hippocampal formation (Hipp), lateral geniculate nucleus (LGN), and dorsal raphé nucleus (DR). Lastly, there were also notable null results: Unlike binding in vole species^18^, we observed no OXTR radioligand binding in the lateral septum (LS) or bed nucleus of the stria terminalis (BNST) of any *Eulemur* specimen (Figs. 2A, 3A). Unlike previous findings in multiple non-human primate species^46,47^, we did not detect OXTR radioligand binding in the nucleus basalis of Meynert.

### AVPR1a distribution across *Eulemur* brains

Relative to OXTR binding patterns in *Eulemur* brains, AVPR1a binding was much more widespread and showed greater average binding across regions (Figs. 2B, 3B,E). In the presence of the OXTR antagonist, we found dense^125^I-LVA binding in specimens across mating systems in the PFC (126.33 ± 17.43) and V1 (116.80 ± 7.92), the Arc (231.13 ± 43.40), along with several areas of the limbic system (LS: 299.54 ± 45.03; BNST: 162.10 ± 13.73; CeA: 170.28 ± 13.38), thalamus (MD 111.70 ± 20.97; medial geniculate [MGN]: 137.80 ± 18.57), and brainstem (periaqueductal gray [PAG]: 119.40 ± 15.77; Sp5: 88.38 ± 18.56). We found moderate binding in the basal ganglia (Cd: 78.20 ± 13.37; Pt: 77.75 ± 11.57; NAcc: 53.76 ± 12.44; GPe: 72.39 ± 16.95; GPi: 64.18 ± 19.73), LGN (88.26 ± 23.38), olfactory cortex (OT: 54.43 ± 12.44; EC: 73.83 ± 17.20; Pir: 39.41 ± 10.24), VMH (79.18 ± 13.56), and other areas of the limbic system (LA: 90.97 ± 7.94; BLA: 67.99 ± 14.23; Hipp: 72.46.31 ± 17.36) and brainstem (SC; 78.80 ± 10.38; SN; 53.23 ± 15.32) (Figs. 2 and 3).

### Binding patterns as a function of mating system

We targeted candidate regions of hypothesized ‘pair-bonding circuits’ in rodents (MeA, NAcc, PFC, LS and BNST;^4^) and titi monkeys (LS, CeA, and Hipp;^49^) in our comparisons of mating system-related differences in *Eulemur* OXTR/AVPR1a binding. Contra Insel and Shapiro’s^18^ consistent findings of greater OXTR binding in monogamous specimens, we found no evidence that OXTR binding patterns in the *Eulemur* amygdala differed significantly between specimens from monogamous vs. non-monogamous species (Figs. 2, 3, 4). We did not observe significant binding in the medial amygdala of any specimens, and in other amygdalar nuclei where OXTR was present, differences between mating systems were non-significant and inconsistent in direction (*d* ranging from − 0.25 to 0.33). Similarly, and somewhat surprisingly, we observed no significant differences in OXTR binding patterns in the NAcc (*d* = 0.13), Hipp (*d* = 0.43), or PFC (*d* = 0.68).

We also observed no statistically significant differences in AVPR1a binding in the regions of interest that were targeted in this analysis for their hypothesized roles in rodent or titi monkey pair-bonding^4,49^. Also divergent from findings in rodents, but consistent with findings in haplorhine primates (e.g.,^47^), we observed no binding in the ventral pallidum of any *Eulemur* specimen. Furthermore, in regions where we observed binding, including the LS, BNST and Hipp, there were no significant differences as a function of mating system. Differences were small to medium for the LS (*d* = 0.41) and BNST (*d* = 0.59), and larger for the Hipp (*d* = 1.03), but all *p* > 0.05 (Figs. 2, 3, 4).

We next examined if there were differences by mating system in any of the regions in which we observed OXTR and/or AVPR1a binding. The only region where we observed significant OXTR differences by mating system was the reticulotegmental nucleus, which showed stronger binding in specimens from monogamous than non-monogamous species (*d* = 3.71, *p* = 0.021; Fig. 4). For AVPR1a, we observed a significant difference in the ventral anterior thalamus (VA; *d* = 1.28, *p* = 0.025), dorsal raphé nucleus (DR; *d* = 1.49, *p* = 0.010), and PFC (*d* = 1.45, *p* = 0.028), with specimens from monogamous species again showing stronger binding in all three regions compared to their counterparts from non-monogamous species (Fig. 4).

## Discussion

As the first study to investigate neuropeptide receptor distribution in strepsirrhine primates, we document binding patterns of both oxytocin and vasopressin in members of the *Eulemur* clade that fall between those of classic rodent models (e.g.^18,19^) and those of more recently characterized haplorhine primates^46,47,57^. This intermediacy may have functional implications for lemurs’ evolutionary specializations, potentially reflecting the comparatively variable role of these neuropeptides in sensory ecology (e.g.^13,58^). As the first primate study to directly compare neuropeptide receptor binding between brain specimens from monogamous and non-monogamous species of the same genus, our findings also fill a critical gap in knowledge of how variation in neuroanatomy reflects variation in primate mating systems or sociality. Beyond simply representing another data point in the domain of comparative neurology, findings from our study of *Eulemur* question the universality of classic vole models and suggest a revisitation of their implications for humans.

Like rodents, lemurs show olfactory specialization^59^, which is prominently displayed in their use of scent to convey a wide array of reproductive and social information^60,61^. Some degree of similarity in the involvement of OXTRs in processing chemically encoded socio-reproductive information in these taxa is suggested by the diffuse binding of both OXTR and AVPR1a in the olfactory bulbs and olfactory tubercle, and by dense binding of AVPR1a in the CeA and BNST (across mating systems). Likewise, AVPR1a binding has been found in the olfactory bulb of platyrrhine primates (e.g. common marmosets;^57^) that also rely extensively on olfactory communication^62^; similar binding has not been reported in less olfactory-oriented catarrhine primates.

Relative to other placental mammals, vision is exceptionally well-developed in primates, but less so in strepsirrhines than in haplorhines. In catarrhines, for example, trichromacy^63,64^ and visual gaze are particularly important in reproductive and social communication^65,66^. Consistent with previous work in haplorhine primates, we found OXTR expression in V1 and the LGN across species, and AVPR1a expression in these and additional areas related to visual attention (i.e., Rtg, SC, and amygdalar nuclei); nevertheless, binding in *Eulemur* was less widespread than that observed in haplorhine primates (e.g.^46,47^). With regard to sensory pathways, therefore, our results are consistent with lemur neuroanatomy representing a bridge between odor-reliant rodents and vision-reliant haplorhines.

Intermediary patterns were also evident in other pathways. For instance, consistent with findings in some rodent species (singing mice:^23^; prairie and montane voles:^19^), but unlike findings in haplorhine primates, we observed dense AVPR1a expression in the MGN of lemurs. Because the MGN is an essential auditory relay nucleus—receiving input from the inferior colliculus and projecting to the auditory cortices—our findings potentially implicate vasopressin in another sensory modality in *Eulemur*; it is possible that vasopressin plays a modulatory role in the processing of vocal communication or emotionally valent sounds.

Relative to haplorhines, additional patterns of receptor binding in *Eulemur* show both similarities and striking reversals. In *Eulemur*, we observed strong AVPR1a binding, but diffuse or modest OXTR binding, in both the striatum and hippocampal regions. This striatal pattern is comparable to that seen in coppery titi monkeys^47^, but it contrasts with the dense OXTR expression found in both rodents^67^ and marmosets^57^. Hippocampal patterns in *Eulemur* are reversed from that observed in titi monkeys^47^. Oxytocin acting on OXTRs in the NAcc is necessary for pair-bond formation in voles^4^. Precise functions of oxytocin or vasopressin within the hippocampal formation remain to be identified^68^, but there is some evidence that they modulate the encoding and consolidation of socially relevant memories^69,70^. In any event, our divergent results in these regions suggest that neural mechanisms of pair bonding in lemurs may differ substantially from other mammalian groups studied thus far.

Regarding the influential hypothesis that interspecific variation in specific populations of receptors reflects variation in social organization or mating system, our results did not reveal comparable differences to the striking findings previously reported for monogamous and non-monogamous vole species. For instance, in Insel and Shapiro^18^, the effect size for a mating system difference in OXTR was *d* = 2.23 in the NAcc and *d* = 2.06 for the LA; in Insel et al.^19^, the mating system difference in AVPR1a was *d* = 3.66 for the LS and *d* = 2.81 for the BNST. In *Eulemur*, despite a sample size that matched these classic vole studies, differences between mating systems, for either neuropeptide, were almost uniformly non-significant (with much smaller effect sizes; all *d* < 0.8) in all regions of a hypothesized rodent ‘pair-bonding circuit’. Our results do not support the suggestion that OXTR/AVPR1a differences in key dopaminergic areas separate monogamous from non-monogamous species^4^. When expanding our comparisons across the entire brain, however, we observed some significant differences between mating systems, including in the Rtp for OXTR and the VA Thal, DR, and PFC for AVPR1a.

How should one interpret these mixed results? Regarding null findings, we note that exhausting the available bank of *Eulemur* brain tissue at the Duke Lemur Center nevertheless left us with limited statistical power to detect differences in individual regions as a function of mating system. While large differences comparable in magnitude to those reported in Insel and Shapiro^18^ and Insel et al.^19^ would be detectable with our sample—indeed, we matched the sample size from these classic studies—more modest differences may have been missed. Regarding exploratory positive findings, we first caution that examining numerous regions increases the potential for false-positives, and that there is a lack of information about the functional significance for many of these differences. For instance, whereas the presence of OXTR in the pontine reticular areas of *Eulemur* and rhesus macaques^46^ suggests a possible conserved function of oxytocin in this region, it is unclear how differences in this region that controls horizontal gaze and saccadic eye movement would be involved in differences in social bonding behavior. Although the ventral anterior thalamus has important functions in spatial memory and learning^71^, it has not been specifically implicated in pair-bonding processes. That said, other findings more readily yield potential interpretations. First, the AVPR1a difference we observed in the DR, a source of serotonin and a region involved in reward-seeking and reward-tracking behavior^72^, suggests that some of the effects of vasopressin on social behavior may owe to activation of the DR serotonin system^73^. If so, monogamous *Eulemur* may have developed denser populations of AVPR1a to bolster serotonergic functions of social reward behavior that foster the creation of pair bonds. Second, rather than observing OXTR binding differences in the PFC—a key area generating the reinforcing, hedonic properties of pair-bonding behavior and mating in rodents^4^—we instead found a difference in AVPR1a binding in this region. Perhaps some of the mechanisms mediated by oxytocin in rodents are carried out by the structurally similar vasopressin in primates—a suggestion that has been hypothesized and substantiated in several previous studies^45^.

Collectively, mixed findings for mating system differences, like the aforementioned binding patterns found across lemur species, are consistent with the existence of distinctive mechanisms for the formation of monogamous mating systems in *Eulemur.* In questioning the universality of these mechanisms across mammalian groups, our findings in this domain can also be considered within the broader context of psychological oxytocin research, which is similarly marked by interpretive challenges and heterogeneous findings (e.g.^74^). We suggest that expanding the toolkits available to researchers, including broadening the animal models studied, will likely continue to reveal unexpected findings that require modification to existing theory (a point echoed by behavioral ecologists; e.g.^75^).

Providing context to our results is the fact that numerous factors other than species identity influence an individual’s oxytocin and vasopressin neurocircuitry. Neurobiology is not static throughout the lifespan, but rather may vary seasonally, with social circumstance, and with age or life-history stage (e.g.^52^). Thus, while receptor distributions can differ widely between species and social systems^18,19,22^, they might also differ substantially *within* individuals of the same species or mating system. Indeed, Phelps and Young^27^ report intraspecific variation in AVPR1a binding among prairie voles often comparable to or greater than interspecific variation (for a recent example of experience-dependent, intraspecific OXTR patterns in a primate model, see^68^). Nevertheless, these same authors also report less variation in regions regulating social bonding, relative to those unrelated to social bonding—a pattern consistent with natural selection winnowing neuropeptide expression in these former regions. We also observed substantial intraspecific and within-mating system variation in *Eulemur* (see individual-level estimates of receptor profiles in Table S3)—given our limited sample size per species, it is unclear to what extent this might be explained by season-level, individual-level, and/or species-level differences. In *Eulemur*, some areas previously identified as key to social bonding—such as nuclei of the amygdala and the BNST—showed relatively small coefficients of variation within mating systems, consistent with^27^, even though they did not differ significantly between mating systems. Other regions that showed relatively little variation within *Eulemur* mating systems, such as the primary visual cortex and SC, were not the same ones identified as part of a pair-bonding circuit in rodent studies, but they are consistently identified as sites of OXTR and AVPR1a in nonhuman primate studies^46,47^. Perhaps neuropeptide binding in regions responsible for processing visual information are important targets of stabilizing selection in primates, regardless of the underlying mating system.

As in the classic vole studies^18,19^, we categorized our *Eulemur* species as belonging to one of two broad mating systems, based on extant information about their wild counterparts^39^. On the one hand, we cannot rule out the possibility that group size reductions, selective reproduction, or long-term pair housing in captivity may have contributed to ‘monogamous-like’ receptor binding profiles across species in our sample, potentially minimizing differences by mating-system category. On the other hand, one might expect such a ‘flattening’ influence to lead to similar receptor profiles across individuals and species, but this does not reflect our results, which are more accurately characterized by a large degree of within-mating system variation. More generally, we believe our results complement the recognition of substantial, natural heterogeneity in social behavior, within or between species, under the general umbrella of ‘monogamous’ or ‘non-monogamous’. Pair-living, pair-bonding, and genetic monogamy are overlapping, yet constitute distinct components of a monogamous mating system that are often conflated^40,76^. Different configurations of these components across ‘monogamous’ species could conceivably create different neuropeptide receptor distributions. Importantly, we note that flexibility in putative mating systems is likely the norm, rather than the exception in animal models. Even the seemingly well-characterized mating system of prairie voles contains surprises revealed only upon extensive observation in naturalistic settings^26^. In some cases, differences in neuropeptide receptor distributions may be detectable in spite of intraspecific (or within-mating system) social variation, but this may less common than previously assumed.

## Conclusion

Our analyses of the oxytocin and vasopressin receptor distributions throughout the *Eulemur* genus break ground into a previously unstudied neurobiological system and question a popular and foundational neurobiological explanation for the differences between monogamous and non-monogamous species. We find in lemurs some elements of neuropeptide expression seen in rodents (e.g. binding in olfactory regions) and other elements more commonly found in haplorhine primates (e.g. binding along visual pathways), consistent with other lines of evidence suggesting the intermediary evolutionary niche occupied by lemurs between other mammalian groups and haplorhine primates. While previous researchers often note the possibility that different mammalian lineages have developed mating systems via distinct neurobiological mechanisms (e.g. ^4^), much of the impact and appeal of rodent studies has come from the enticing possibility that conserved mechanisms related to oxytocin and vasopressin may help explain how human pair bonds are formed. We show that circuits identified as key to pair bonding in rodents cannot simply be invoked to explain primate pair bonding. Our research on the lemur oxytocin system, as part of burgeoning body of work across a range of nonhuman primates, also has important implications for translational research, as it provides a glimpse into the diversity by which these neuropeptides may have their manifold effects on social behavior.

## Supplementary Information

Supplementary Information 1.

## Data Availability

The data and corresponding R code needed to reproduce our results are publicly available at https://osf.io/rymz5/.

## Abbreviations

Arc: Arcuate nucleus
BLA: Basolateral amygdala
BNST: Bed nucleus of the stria terminalis
CeA: Central amygdala
Cd: Caudate
DG: Dentate gyrus
DR: Dorsal raphe nucleus
EC: Entorhinal cortex
GPe: Globus pallidus external segment
GPi: Globus pallidus internal segment
Hipp: Hippocampal formation
IO: Inferior olive
LA: Lateral amygdala
LGN: Lateral geniculate nucleus
LS: Lateral septum
MD: Medial dorsal thalamus
MGN: Medial geniculate nucleus
NAcc: Nucleus accumbens
NP: Nucleus prepositus
OB: Olfactory bulb
OT: Olfactory tubercle
PAG: Periaqueductal gray
PFC: Prefrontal cortex
Pir: Piriform cortex
Pt: Putamen
PVNth: Paraventricular nucleus of the thalamus
Rtg/Rtp: Reticulotegmental/reticulopontine nucleus
SC: Superior colliculus
SN: Substantia nigra
V1: Primary visual cortex
VA: Ventral anterior thalamus
VMH: Ventromedial hypothalamus
