# Supplementary material for: Neural correlates of mating system diversity: oxytocin and vasopressin receptor distributions in monogamous and non-monogamous Eulemur
Source: Sci Rep. 2021 Feb 12;11:3746. doi: 10.1038/s41598-021-83342-6 (PMC7881006; doi:10.1038/s41598-021-83342-6)
Supplement: Supplementary file 1 — Supplementary Information 1. [file 41598_2021_83342_MOESM1_ESM.pdf]

Supplementary Online Materials for *"Neural correlates of mating system diversity: oxytocin and vasopressin receptor distributions in monogamous and non-monogamous Eulemur"*

Authors: Nicholas M. Grebe, Annika Sharma, Sara M. Freeman, Michelle C. Palumbo, Heather B. Patisaul, Karen L. Bales, & Christine M. Drea

Table S1. Quantification of competitive displacement of the ^{125}I-OVTA radioligand by antagonists for both OXTR and AVPR1a. Estimated dpm/mg (mean ± SEM) are reported for four representative brain regions, and for the average across all regions, for each binding condition. Two-tailed paired *t*-tests revealed a significant reduction in binding by the vasopressin 1a receptor (AVPR1a) antagonist (+SR49059) compared to radioligand alone in three of the four regions, as well as overall, indicating that the OXTR radioligand binds nonspecifically to AVPR1a. The oxytocin receptor (OXTR) antagonist (+ALS-II-69) significantly reduced binding compared to radioligand alone in all four regions, as well as overall, indicating that the OXTR radioligand binds to OXTR. Abbreviations: CeA, central amygdala; NAcc, nucleus accumbens; Sp5, spinal trigeminal nucleus; V1, primary visual cortex.

Brain Regions	Optical binding densities (mean ± SEM)			Two-tailed paired *t*-tests	
	^{125}I-OVTA Alone	^{125}I-OVTA + SR49059	^{125}I-OVTA + ALS-II-69	Alone vs. +SR49059	Alone vs. +ALS-II-69
CeA	42.03 ± 10.21	13.95 ± 3.97	15.48 ± 6.44	$t_8 = 4.192$; $p = 0.003$	$t_8 = 3.019$; $p = 0.017$

Brain Regions	Optical binding densities (mean ± SEM)			Two-tailed paired *t*-tests	
	**^{125}I-OVTA Alone**	**^{125}I-OVTA + SR49059**	**^{125}I-OVTA + ALS-II-69**	**Alone vs. +SR49059**	**Alone vs. +ALS-II-69**
NAcc	32.09 ± 5.95	19.20 ±6.31	16.22 ± 4.80	$t_9 = 2.449$; $p = 0.037$	$t_9 = 1.90$; $p = 0.090$
Sp5	60.13 ± 10.97	43.86 ± 9.51	2.51 ± 1.29	$t_7 = 3.862$; $p = 0.006$	$t_7 = 5.532$; $p < 0.001$
V1	107.85 ± 21.37	86.66 ± 17.33	38.89 ± 8.40	$t_{10} = 2.107$; $p = 0.061$	$t_{10} = 3.503$; $p = 0.006$
Overall average	71.51 ± 6.44	55.02 ± 6.44	20.90 ± 2.11	$t_{216} = 7.97$; $p < 0.001$	$t_{210} = 10.125$; $p < 0.001$

Table S2. Quantification of competitive displacement of the ^{125}I-LVA radioligand by antagonists for AVPR1a and OXTR. Estimated dpm/mg (mean ± SEM) are reported for four representative brain regions, and for the average across all regions, for each binding condition. Two-tailed paired *t*-tests revealed a significant reduction in binding by the vasopressin 1a receptor (AVPR1a) antagonist (+SR49059) compared to radioligand alone in three out of the four regions, as well as overall, indicating that the AVPR1a radioligand binds to AVPR1a. The oxytocin receptor (OXTR) antagonist (+ALS-II-69) did not significantly change binding compared to radioligand alone in any of the four regions, but there was a significant reduction in binding when all measured regions were averaged, indicating that the AVPR1a radioligand is specific to AVPR1a, but does show some

nonspecific binding to OXTR. Abbreviations: CeA, central amygdala; NAcc, nucleus accumbens; Sp5, spinal trigeminal nucleus; V1, primary visual cortex.

Brain Regions	Optical binding densities (mean ± SEM)			Two-tailed paired *t*-tests	
	**^{125}I-LVA Alone**	**^{125}I-LVA + SR49059**	**^{125}I-LVA + ALS-II-69**	**Alone vs. +SR49059**	**Alone vs. +ALS-II-69**
CeA	195.16 ± 20.59	70.90 ± 16.95	170.28 ± 13.38	$t_{10} = 4.348$; $p = 0.001$	$t_{10} = 1.286$; $p = 0.228$
NAcc	58.25 ± 8.23	40.72 ± 12.05	53.76 ± 12.57	$t_{10} = 2.095$; $p = 0.060$	$t_{10} = 0.310$; $p = 0.763$
Sp5	95.84 ± 21.82	42.81 ± 9.61	88.38 ± 18.56	$t_7 = 3.802$; $p = 0.007$	$t_7 = 1.130$; $p = 0.296$
V1	120.27 ± 14.86	90.96 ± 15.62	116.80 ± 13.31	$t_{11} = 2.744$; $p = 0.019$	$t_{11} = 0.375$; $p = 0.715$
Overall average	113.77 ± 5.16	52.01 ± 2.52	102.01 ± 4.51	$t_{318} = 13.422$; $p < 0.001$	$t_{314} = 4.548$; $p < 0.001$

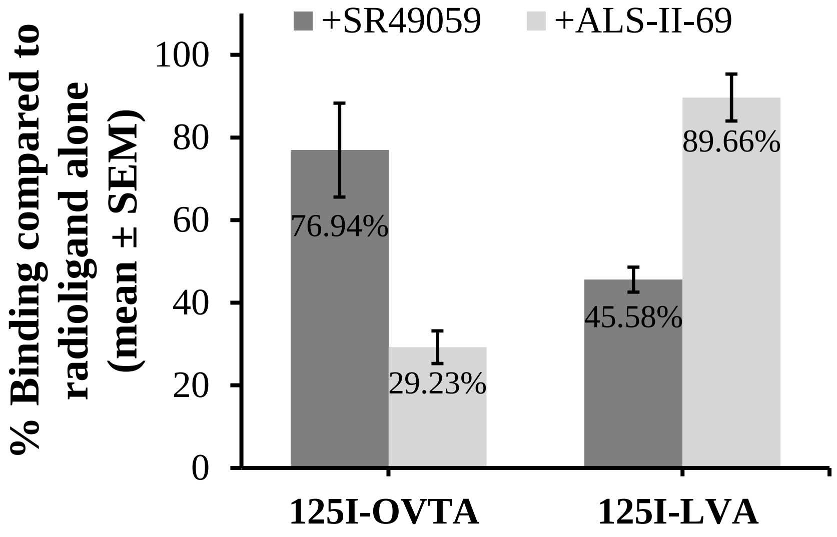

Fig. S1. Overall efficacy of the small molecule antagonists for displacing radioligand binding. Bars represent the percent reduction in radioligand binding (±SEM) by SR49059 and ALS-II-69, averaging across all brain regions quantified. ALS-II-69 displaces an average of 70.77% of ^{125}I-OVTA, but only 10.34% of ^{125}I-LVA. SR49059 displaces 54.42% of ^{125}I-LVA, but only 23.06% of ^{125}I-OVTA.

Table S3. Estimated OXTR/AVPR1a binding (dpm/mg; average of three replicates) in key regions of interest, by individual specimen. This simplified table only lists estimated density of receptors in a subset of all regions measured; a complete quantitative dataset is publicly available at https://osf.io/rymz5/.

**Animal**	Dido	Paiute	Fabio[1]	Moheli	Deucalion	Teucer	Harlow	Lamour	Fiery	Frigga	Francoise[2]	Jules[2]
**Species**	*rubriventer*	*rubriventer*	*mongoz*	*mongoz*	*macaco*	*macaco*	*flavifrons*	*flavifrons*	*rufifrons*	*fulvus*	*collaris*	*collaris*
**Sex**	F	M	M	F	M	M	F	F	M	F	F	M
**Mating**	mono	mono	mono	mono	nonmono	nonmono	nonmono	nonmono	nonmono	nonmono	nonmono	nonmono
						**OXTR**						
PFC	76.85	18.80	148.58	122.27	64.90	105.97	61.05	82.51	46.23	41.00	+	+
NAcc	9.83	0.00	62.47	10.55	0.24	29.85	15.46	33.06	0	30.58	+	+
BLA	44.00	7.89		9.40	15.89	22.98	12.94	18.33	51.74	26.39		
CeA	20.40	0.71		3.46	6.25	12.79	13.75	8.87	40.29	19.03		
LA	47.21	10.54		18.22	12.16		26.22	33.98	59.27	25.18		
						**AVPR1a**						
LS	567.19	170.85		280.07	128.84	389.03		202.73	355.87	175.19	260.86	464.81
BNST	181.26	190.91		177.95	150.16	138.25	139.47	215.92	246.01	135.25	121.91	86.06
BLA	95.72	100.35		57.21	72.11	189.01	32.00	24.77	40.34	48.31	54.23	33.85
CeA	252.25	212.39		156.56	137.29	167.90	144.32	184.23	220.45	170.56	106.25	120.95
LA	106.54	110.12		33.17	99.64	190.46	42.87	70.06	67.09	75.50	99.76	105.46
Hipp	169.62	82.80		75.61	47.83	35.19	0	26.48	161.73	67.12	58.20	

[1] Most midline structures were unquantifiable for this specimen due to tissue damage.

[2] For OXTR in these specimens, a failed competitive binding condition (^{125}I-OVTA + SR49059) prevented quantitative binding determination. However, the ^{125}I-OVTA alone condition was successful, allowing for a qualitative assessment of presence (+) or absence of OXTR in these regions.
